# Graphene Nanoplatelet- and Hydroxyapatite-Doped Supramolecular Electrospun Fibers as Potential Materials for Tissue Engineering and Cell Culture

**DOI:** 10.3390/ijms20071674

**Published:** 2019-04-03

**Authors:** Vassilis Kostopoulos, Athanasios Kotrotsos, Kalliopi Fouriki

**Affiliations:** 1 Department of Mechanical Engineering and Aeronautics, University of Patras, Patras University Campus, GR-26504 Patras, Greece; akotrotso@mech.upatras.gr (A.K.); lfouriki@gmail.com (K.F.); 2 Foundation of Research and Technology, Institute of Chemical Engineering Sciences (FORTH/ICE-HT), Stadiou Str., GR-26504 Patras, Greece

**Keywords:** supramolecular polymers, hydrogen bonds, electrospinning, GNPs, hydroxyapatite, scaffolds, tissue engineering, cell culture

## Abstract

Porous and fibrous artificial extracellular matrices (ECM) called scaffolds are considered to be promising avenues of research in the field of biomedical engineering, including tissue fabrication through cell culture. The current work deals with the fabrication of new matrix-type scaffolds through electrospinning, in order to support future three-dimensional tissue formation. The selected material for the fabrication of these scaffolds was a supramolecular polymer (SP) that is based on ureiodypyrimidone hydrogen bonding units (UPy). More precisely, pure SP and modified electrospun scaffolds with (a) graphene nanoplatelets (GNPs), (b) hydroxyapatite (HA), and (c) a mixture of both were fabricated for the needs of the current study. The aim of this work is to engineer and to characterize SP electrospun scaffolds (with and without fillers) and study whether the introduction of the fillers improve the physical and mechanical properties of them. The obtained results indicate that doping the SP scaffolds with GNPs led to improved apparent mechanical properties while HA seems to slightly deteriorate them. For all cases, doping provided thinner fibers with a more hydrophilic surface. Taking together, these types of SP scaffolds can be further studied as potential candidate for cell culture.

## 1. Introduction

Solution electrospinning is a simple, inexpensive, versatile, and cutting-edge technique for the fabrication of nonwoven fibrous materials at the nano- and microscale (fibers with tunable diameter, from 10 μm to 10 nm) [1]. During the electrospinning process, an electrified needle ejects a polymeric solution towards a grounded collector. When a large electric field is applied, a one-dimensional structure is created by the solution and the solvent evaporates as the electrified jet travels from the end of the needle toward the collector to produce solid polymeric fibers [1]. A typical electrospinning setup consists of a power supply, a solution reservoir connected with a nozzle, and a collector [2,3]. By utilizing the electrospinning method, fibers with different morphological, physical, and mechanical properties can be obtained. These tunable properties depend on the different process parameters applied to the system (i.e., applied voltage, solution flow rate, distance between the tip of the nozzle with the collector, etc.) [4].

In the past few years, extensive work has been carried out on electrospinning, and it has shown a great potential in the fields of filtration, batteries, composite materials, and especially biomedicine [2,3,4,5,6]. Apart from electrospinning method, in biomedicine, a variety of methods have been utilized for the fabrication of scaffolds with various architectural configurations and geometries, in order to mimic ECM. These methods involve melt extrusion, rapid prototyping, and solvent evaporation [7,8,9,10,11,12]. Of these, electrospinning has attracted the interest of researchers as a simple and effective method because the electrospun fabricated scaffolds are highly porous with high specific surface area and have ECM-like nanotopography. Many investigations reported scaffold fabrication of synthetic and biodegradable polymers such as poly(lactic-co-glycolicacid) (PLGA) by electrospinning [13]. Wang et al. [14], combined PLGA with other biomaterials, while Scaffaro et al. in [15], subjected the same material to O_2_ plasma treatment.

The mechanical properties and the hydrophilicity of a scaffold play an important role on its in vivo performance in tissue engineering. In general, implanted scaffolds are typically subjected to stresses (i.e., tension, compression, torsion, shearing, etc.), and not all the materials used to fabricate scaffolds are strong enough to mimic native tissues [16]. Over the past decades, extensive research has been conducted with an aim to improve the mechanical performance and hydrophilicity of the artificial ECM. Incredible improvements have been reported in literature by the incorporation of HA and GNP particles within the fabricated scaffold [16,17,18,19,20,21,22,23,24]. HA is one of the main component minerals of bone and teeth and has favorable biocompatibility. Its excellent mechanical properties make this ceramic material a potential biomaterial for bone regenerative medicine [16]. The chemical and structural similarity of HA to the mineral phase of a native bone makes it to show an appropriate osteoconductivity and biocompatibility [16,17,18]. On the other hand, GNPs and their derivatives (i.e., graphene oxide) have received increasing attention for biomedical applications because they exhibit remarkable properties (i.e., high surface area, high mechanical strength, ease of functionalization, etc.). In addition, GNPs can promote and enhance osteogenic differentiation making it a potential material for bone regeneration domain [19,20,21,22,23,24]. GNPs can be obtained by using different methods. These methods involve mechanical and/or chemical exfoliation of graphite [21,25,26].

Conventionally, based on the literature, the solution electrospinning technique is utilized to electrospun covalent polymers with large molecular weight, but has recently extended to supramolecular assemblies (i.e., surfactants, peptides, cyclodextrin, host–guest complexes, etc.) [27,28,29]. Yan et al. [30], produced supramolecular polymer (SP) nanofibers by electrospinning of a heteroditopic monomer that is based on a crown ether ammonium salt interaction. Hermida-Merino et al. [31], successfully fabricated SP electrospun fibers of low molecular weight self-assembling polyurethanes and investigated the relationship between the hydrogen bonding strength of the end group and the ability to produce fibers. Tayi et al. in [32], reported the electrospinning of functional peptide-based SP from water at very low concentrations (<4 wt %). Peptides are a class of self-assembling biomolecules and have been extensively investigated for a wide range of biomedical applications such as spinal cord injury, wound healing, and enamel [33,34,35,36].

Based on that, a new technology that could be beneficial for tissue engineering and cell culture has been built on SP [37,38,39]. Especially those based on reversible hydrogen bonding arrays (noncovalent interactions) [40,41,42,43], show great promise for biomedicine and tissue engineering, since these materials provide numerous advantages. These advantages involve (a) SPs’ small molecules that help to reproducibly synthesize them and (b) self-assembled behavior that allows them to be morphologically well-defined. In such self-assembled molecular compounds, the polymer chains often act as a host for the guest small molecules to form a clathrate [44]. In the present study, we have employed the ureiodypyrimidone hydrogen bonding unit (UPy) as developed by Meijer and coworkers [45] because of its strong self-association, its synthetic accessibility, and the highly dynamic nature of low glass transition temperature (Tg) polymers comprising the UPy [45]. Most interestingly, UPy polymers have recently been shown to give unprecedented toughening in polybutadiene-based interpenetrating networks [46]. This type of SP was also recently incorporated into carbon fiber-reinforced plastics as interleaves, in order to play the self-healing agent role [47,48,49]. Experimental results showed not only the enhancement of the mechanical properties but also a high recovery of them was obtained after fracture.

The scope of the present work is the use of a new, functional and flexible low molecular weight SP comprising UPy moieties to produce bioactive fibers and matrices for tissue fabrication through cell culture. More precisely, (a) GNPs, (b) HA, and (c) mixture of both, were employed to modify hydrogen-bonded SP electrospun scaffolds that are based on UPy units. The effect of these fillers on the fibers’ structural, physical and mechanical properties is extensively assessed. In future work, hydrogen-bonded SP scaffolds (with and without fillers) can be further studied as potential candidate for cell culture with an aim to fabricate a tissue for biomedical applications.

## 2. Materials and Methods

### 2.1. Raw Materials

The SP was developed and supplied by Suprapolix. The supplied material (Batch identification code: SPSH01) is based on a low Tg (−66 °C) polymer modified with UPy moieties, and was chosen for the needs of the present study. This polymer owes its physical and mechanical properties to the reversible, noncovalent interactions, such as hydrogen bonding, between the macromolecules. Formic acid (FA), which played the solvent role, was purchased from Sigma-Aldrich (reagent grade ≥ 95%). Regarding the nanofillers, the four-layered GNPs were supplied by Cheap Tubes Inc., Cambridgeport, VT, USA having lateral dimensions of 1–2 μm, average thickness lower than 4 nm, surface area higher than 750 m^2^/gr, and purity higher than 99%. The synthetic “needle-like” HA nanoparticles that were utilized was purchased by Shanghai Xinglu Chemical Tech., Shanghai, China, having an average length of 150 nm, an average thickness of 20 nm, and purity higher than 97.5%. In addition, HA nanoparticles contain many minerals (Mg ≤ 1.8%, Na ≤ 0.2%, Fe ≤ 0.08%, and Al ≤ 0.1%) that are expected to increase the electrical conductivity of the solution’s prior electrospinning process.

### 2.2. Solution Preparation and Electrospinning

SP, GNPs, and HA were dissolved in FA. The total concentration of all materials in the solution was 15 wt%. Four different solutions were created, one with pure SP, one with 0.075 wt% GNPs (0.5 wt% into the final scaffold), one with 0.15 wt% HA (1 wt% into the final scaffold), and a fourth one with 0.075 wt% GNPs, together with 0.15% wt% HA (0.5 wt% GNPs and 1 wt% HA into the final scaffold). All blend solutions were constantly stirred for 48 h at room temperature (RT) in order to achieve homogeneity. Each solution was transferred into 3 mL plastic syringe for electrospinning. The solution flow rate for all samples was kept stable at 0.02 mL/h in order to achieve a stable process. The applied voltage was kept constant at 18 kV in order to achieve continuous jet formation. The distance between the tip of the nozzle and the collector was kept constant at 15 cm. Disposable blunt-tipped needles (Howard Electronic Instruments, HEI) of an inner diameter of 0.6 mm were used. A grounded, square aluminum collector (15 × 15 cm^2^) was used to collect the scaffolds. Electrospinning was performed at RT. The scaffolds were removed from the collector and left to dry for 24 h at RT.

### 2.3. Electrical Conductivity

The electrical conductivity of all the different solutions with and without fillers was measured using a conductometer (Sension EC5 Portable Conductivity/TDS Meter). 20 mL of each solution were measured at RT.

### 2.4. Scanning Electron Microscopy and Transmission Electron Microscopy

Square strips of 5 × 5 mm^2^ were punched out from each electrospun specimen and were sputter-coated with gold for 30 s, before being placed inside the field emission scanning electron microscopy instrument (FE–SEM, FEI Inspect TM F50) by using a scanning electron (SE) detector. The signal by the backscattered electrons is collected by the SE detector and has been converted to a signal that is sent to a computer for further analysis. The images were obtained, using magnitudes ranging from ~0.3k× to ~20k×.

On the other hand, circular carbon-coated copper grids (200 mesh) with diameter 3.05 mm were utilized for the transmission electron microscopy (TEM) characterization experiments for all scaffold types (both pure and modified ones). These grids were positioned carefully on the collector’s surface in order for the electrospun fibers to be deposited during the electrospinning process. The TEM instrument model was the JEM-2100/ HR-TEM, was produced by Japan Electron Optics Laboratory (JEOL) and operated at 200 kV.

Fiber diameter measurements were obtained by image processing with ImageJ software (NIH, Bethesda, Maryland, USA). At least 150 fiber diameters were measured while the average value and standard deviation (SSD) were reported.

### 2.5. Porosimetry and Porosity

The average pore area for pure and modified SP scaffolds was also determined by image processing of SEM images with ImageJ software (NIH, -Bethesda, Maryland, USA). In addition, the scaffold’s porosity was determined from the ratio of the measured mass of the sample to the mass of a fully dense sample of the same size by measuring the sample’s dimensions (i.e., length, width, and thickness). The thickness of all scaffolds was measured with a thickness gauge by applying constant force. The porosity was determined by using the Equation (1) below.

(1) $$P=\frac{M_{1}-M_{2}}{M_{1}}\cdot 100\;(\%)$$

where P is the porosity, M_1_ is the mass of a fully compacted sample, and M_2_ is the mass of an electrospun scaffold. All utilized samples had the same dimensions for comparison reasons.

### 2.6. Static Water Contact Angle Assay

Square strips of 5 × 5 mm^2^ were punched out from each specimen to investigate the contact angles of water droplets on the surface of the fiber mats, using an optical contact angle goniometer apparatus (Kruss DSA100, Hamburg, Germany). Measurements were taken at *t* = 0 s after a single droplet of bi distilled water (2 μL) got in contact with the surface of the fiber scaffolds. Five measurements were taken for each sample and the experiments were performed at RT.

### 2.7. Tensile Tests

Five rectangular samples of 30-mm-length and 10-mm-width were punched out from each scaffold. The thickness of each specimen was measured with a thickness gauge by applying constant force. Duct tape was carefully placed at each edge of the samples to improve the mounting on the metallic grips of the testing device. Uniaxial tensile tests were conducted on a Minimat 2000 tensile instrument with a 200 N load cell (Rheometric Scientific, Shakopee, MN, USA). During the experiments the span length was 12 mm. The Young’s modulus (E), the ultimate tensile strength (σ_max_), and the elongation at break (ε_max_) % (apparent mechanical properties) were measured and analyzed. All experiments were performed at RT until sample failure at a strain rate of 5 mm/min.

## 3. Results and Discussion

### 3.1. Structural and Morphological Analysis

**Figure 1:**
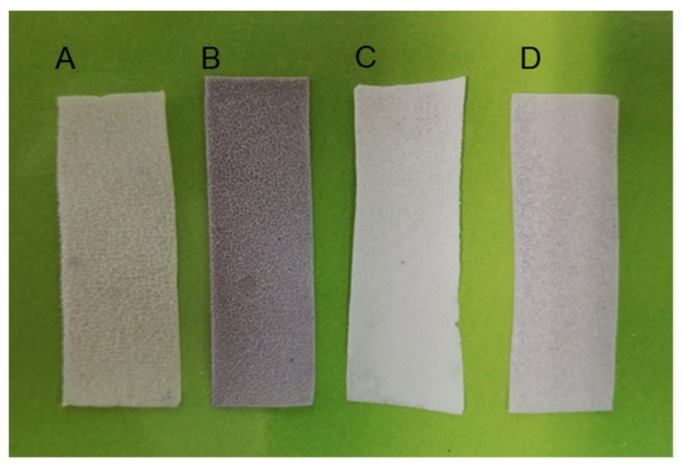
Illustration of the fabricated scaffolds utilized for the needs of the current study: (**A**) pure supramolecular polymer (SP), (**B**) SP modified with graphene nanoplatelets (GNPs), (**C**) SP modified with hydroxyapatite (HA), and (**D**) SP modified with GNPs and HA.

The structure and morphology of all SP scaffold types (pure and modified ones with GNPs, HA, and mixture of them) were characterized using SEM and TEM microscopies. In Figure 1, is clearly illustrated scaffolds’ final form after fabrication by electrospinning. According to this figure, all scaffolds seem to be uniform and homogeneous. The characteristic dark gray color for the sample that is illustrated in Figure 1B is attributed to the strong presence of the GNPs at the amount of 0.5 wt%. On the other hand, samples containing 1 wt% HA (see Figure 1C) seem to be whiter if compared to the reference one (see Figure 1A) due to the strong presence of the HA nanofillers (1 wt%). Finally, sample containing the mixture of both fillers (GNPs and HA) seems to obtain a lighter gray color if compared to the Figure 1B due to the high content of the HA nanofillers (1 wt%).

**Figure 2:**
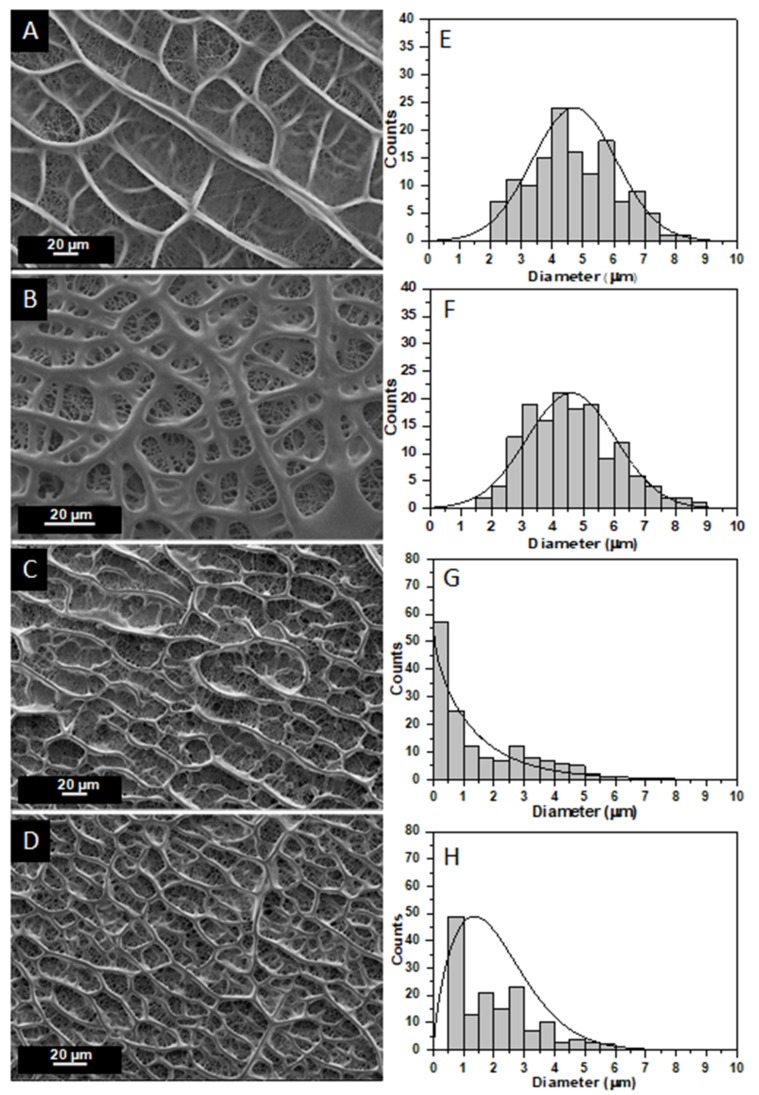
SEM images of (**A**) pure SP scaffold, (**B**) modified SP scaffold with GNPs, (**C**) modified SP scaffold with HA, and (**D**) modified SP scaffold with GNPs and HA. Histogram providing the fiber diameter distribution for (**E**) pure SP scaffold, (**F**) modified SP scaffold with GNPs, (**G**) modified SP scaffold with HA, and (**H**) modified SP scaffold with GNPs and HA.

**Figure 3:**
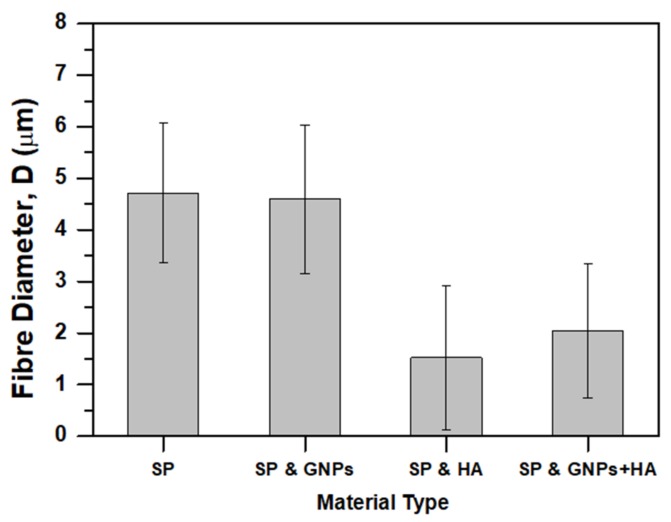
Bar chart diagram providing average fiber diameters and standard deviations (SSD) for all scaffold types (pure SP and modified SP with GNPs, HA, and mixture of them).

Figure 2 shows SEM images of the scaffolds (pure and modified ones) fabricated, together with the histograms that illustrate the scaffolds’ fiber diameter distribution. Figure 2A–D correspond to the pure SP, 0.5 wt% GNP-modified, 1 wt% HA-modified, and 0.5 wt% GNP with 1 wt% HA-modified scaffolds, respectively. According to these images, no fibers with beads were detected on the surface of all electrospun scaffold types. In all cases, smooth, cylindrical beadless fibers were fabricated consisting of dense networks with no particular alignment (especially the modified ones). Also, these images show a well interconnected pore network structure. The average fiber diameter and distribution of values are depicted in Figure 2E–H, respectively. Figure 3 summarizes the average fiber diameters for all scaffold types together with standard deviations. Taking into consideration these histograms of Figure 2 and the bar chart diagram of Figure 3, it was shown that the average fiber diameter was generally decreased by modification with scaffolds containing HA to exhibit the lower values. More precisely, the average fiber diameter was decreased from 4.71 ± 1.36 μm with the absence of fillers to 4.59 ± 1.44 μm for samples containing 0.5 wt% GNPs, to 1.52 ± 1.48 μm for samples containing 1 wt% HA and to 2.04 ± 1.29 μm for samples containing 0.5 wt% GNPs and 1 wt% HA. Values obtained for pure and modified SP with GNPs exhibited normal distribution while the other ones containing HA differed significantly to each other as exhibited non-normal (Weibull) distribution. According to these experimental results, it was shown by solution modification with fillers (GNP and HA) that the final fiber tends to be thinner with HA-modified ones to exhibit the thinner ones. HA seems to have a strong impact on final scaffold’s fiber diameter as affected the fiber diameter values distribution. An analogous behavior was also observed in Gkermpoura’s et al. [26] and in Tyagi’s et al. [50] works, in which the authors altered the solution’s properties by GNPs and HA modification, respectively.

It can be expected that the addition of both filler types (GNPs or/and HA) in the solution prior electrospinning process altered the solution parameters (i.e., electrical conductivity) [4]. Electrical conductivity measurements have been conducted and will be thoroughly discussed in the next paragraph (3.2), in which the physical characterization is extensively discussed. The reduction of the fiber diameter for all modified scaffolds could be attributed to the presence of the GNP and HA molecules inside the polymeric solutions that contribute to electrostatic charge build up during electrospinning. As the jet exits the tip of the nozzle, it reinforces the effect of the electric field to obtain thinner fibers. In addition, the fillers possibly led to lowering of the surface tension of the polymeric solution as a result the bending instability to be enhanced during the phenomenon. According to relative literature, GNPs have the potential to increase the electrical conductivity [20,21]. The utilized synthetic HA nanofillers contain many minerals as previously reported (see paragraph 2.1) and if take into consideration the dissolution of the HA into FA produces ions, the electrical conductivity of the solution is expected to increase to a great extent.

**Figure 4:**
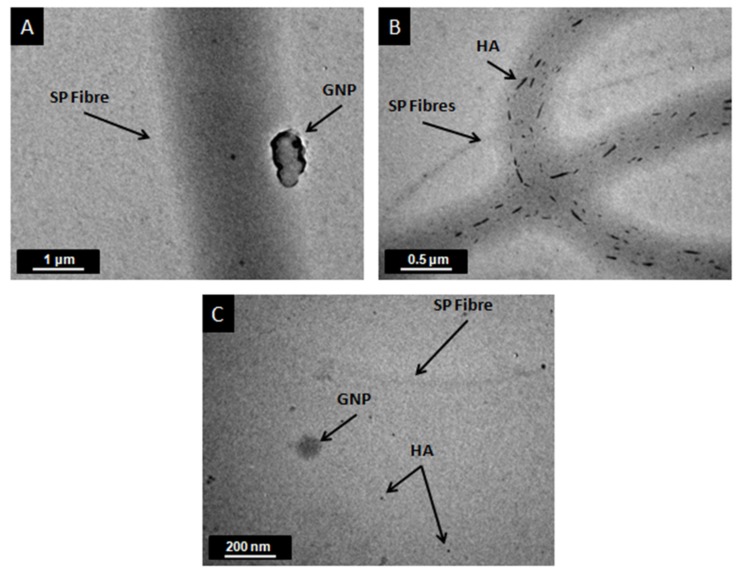
TEM images of (**A**) modified SP scaffold with GNPs, (**B**) modified SP scaffold with HA, and (**C**) modified SP scaffold with GNPs and HA.

To investigate the internal structure of GNP and HA fillers into SP fibers, all modified SP scaffolds were characterized by TEM microscopy. The electrospun fibers prior TEM were collected onto the surface of copper grids as previously described in paragraph 2.4. In Figure 4A–C, the incorporation of GNPs (Figure 4A), HA (Figure 4B), and the mixture of them (Figure 4C) into the fibers is shown. For all scaffold types, the fillers are oriented along the axial direction of the fibers. This behavior is attributed to the stresses imparted on the electrospun fibers during the electrospinning process that led to filler alignment along the fiber axis. An analogous behavior was also observed in [6,26], in which the fibers were doped by multiwall carbon nanotubes (MWCNTs) and GNPs respectively. In addition, these images validated the fiber diameter values, which were calculated by SEM images, as well as the trend of the fiber diameter to decrease by modification.

### 3.2. Physical Characterization

The addition of the GNPs and HA into polymeric solutions is expected to have an impact on solutions’ electrical conductivities and static water contact angles’ assay values. This section thoroughly investigates the effect of the solution modification on both properties.

**Figure 5:**
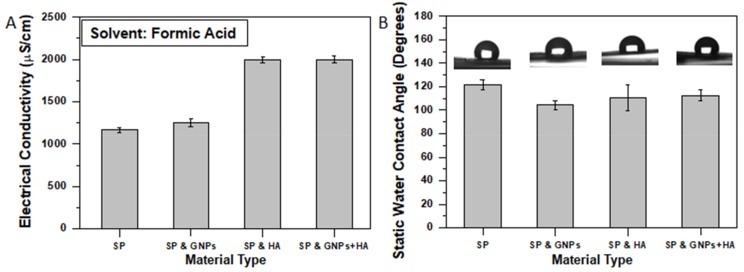
Bar chart diagrams providing (**A**) the solutions’ electrical conductivities prior electrospinning process and (**B**) the influence of filler type on the average static contact angle of distilled water droplets formed on samples’ surface (at *t* = 0 sec). For both bar chart diagrams standard deviations (SSD) are provided.

As previously reported (see paragraph 3.1), with modification the final fiber, the diameter of the scaffolds has been significantly affected (mostly for the HA doped ones). By increasing the electrical conductivity of a solution leads to the enhancement of the bending instability of the ejected polymeric solution during the electrospinning process. Thus, the elongation of the jet increases and consequently results in thinner fibers. The electrical conductivity of the SP polymeric solutions was increased from 1169 ± 30.12 μS/cm in the absence of fillers to 1255 ± 47.84 μS/cm for samples containing 0.5 wt% GNPs to 2000 ± 36.52 μS/cm for samples containing 1 wt% HA and to 2000 ± 42.87 μS/cm for samples containing 0.5 wt% GNPs and 1 wt% HA (see Figure 5A).

The significant increase of the electrical conductivity values is attributed to the sufficient transfer of the charges into the solution [3]. For solutions containing HA the electrical conductivity was almost doubled. According to relative literature [51], this behavior is not only attributed to the containing minerals (as previously reported) but also to the chemical reaction mechanism of the HA with the FA. In this mechanism, H^+^ ions (from the acid) are defined as the primary crystal lattice-disrupting agent. Also, H^+^ ions in the solution approach the apatite crystal and react with PO_4_^3−^ and HO^−^ ions at the surface of the solid. Conversion to HPO_4_^2−^ and H_2_O causes a disruption of lattice bonds and release of the ions into the solution. This phenomenon appears frequently in the literature and can be identified as “acid reactivity”, “acid attack”, and “chemical reactivity” [51].

A static contact angle assay was also conducted to investigate the effect of the fillers on the hydrophilicity of the SP fibrous scaffolds. According to experimental results, it was shown that by modification of the pure SP scaffold with fillers (GNPs, HA, and mixture of them) the hydrophilicity of the scaffolds’ surface increased as the measured contact angle values decrease. More precisely, the average static water contact angle for pure SP scaffolds (reference material) was 121.76 ± 4.13^0^, while for modified ones was 104.52 ± 3.80^0^ (14.1% decrease) for samples containing GNPs, 110.56 ± 11.14^0^ (9.1% decrease) for samples containing HA, and 112.68 ± 4.67^0^ (9.2% decrease) for samples containing GNPs and HA. These results are in line with other investigations, in which polymeric scaffolds increase its hydrophilicity by modification [6,52].

### 3.3. Porosimetry and Porosity

**Figure 6:**
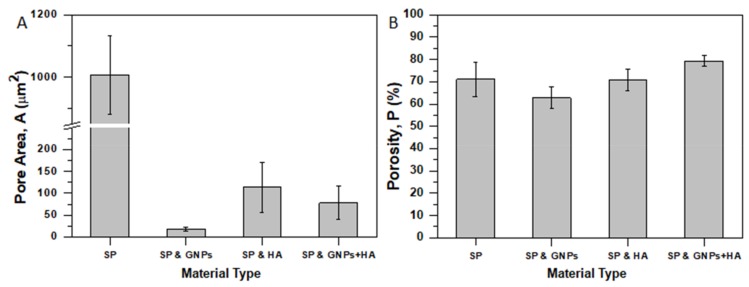
Effect of filler type (**A**) on scaffolds’ pore area (**A**) and (**B**) on scaffolds’ porosity (P), together with standard deviations (SSD).

Bar charts of Figure 6 show the pore area (Figure 6A) and porosity (Figure 6B) values with respect to material type. This section thoroughly investigates the influence of the filler type on the pore sizes and porogen fraction to the final scaffold.

According to SEM images of Figure 2, generally the pore area significantly decreases by modification with samples containing 0.5% GNPs to exhibit the lower values. The bar chart of Figure 6A summarizes the pore area values for all material sets. For the pure SP scaffolds, the pore area value was calculated to be 1006 ± 126 μm^2^ (the higher one), for the GNP-modified 17 ± 4 μm^2^ (98.3% reduction), for the HA-modified 113 ± 57 μm^2^ (88.7% reduction), and for the mixture of fillers 77 ± 38 μm^2^ (92.3% reduction). Based on these results, it was shown that both GNPs and HA (especially GNPs) fillers significantly affect the final pore area as the pore shrinks greatly. Additionally, the experimental results suggest that the HA presence tends to increase the pore area.

The bar chart of Figure 6B shows the apparent porosities for all scaffold types. The porosity of the reference scaffold (pure SP) was calculated to be 71.1 ± 7.8%, for the GNP-modified one 62.7 ± 4.9%, for the HA-modified one 70.7 ± 4.8%, and for the scaffold containing both GNPs and HA 79.3 ± 2.2%. These results suggest that by GNP modification the porosity of the final scaffold decreases by 9%, the HA presence does not affect the porogen fraction, while the fillers’ mixture enhance the porosity by 9%. The obtained results are in line with average pore area described above. In the case of GNP-modified scaffolds, the pore area significantly decreases by almost 98% together with the slight decrease of the average fiber diameter, resulting in a decrease of the final’s scaffold porosity. On the other hand, scaffolds containing HA exhibited extremely decreased average fiber diameter and pore area that led these samples to exhibit comparable (HA-modified samples) or slightly increased (GNP and HA-modified samples) porogen fraction.

### 3.4. Mechanical Properties

The apparent mechanical properties of the fabricated porous SP electrospun scaffolds (reference and modified ones) were investigated by conducting uniaxial tensile tests. The incorporation of the fillers is also expected to affect the mechanical behavior of the final scaffold. Tensile tests were conducted according to specifications described in paragraph 2.7. Rectangular specimens were machined from the fibrous electrospun mats and mounted on testing cards. In order to avoid sever damage, special care was taken during the preparation process and then the specimens were gripping to the tensile apparatus.

**Figure 7:**
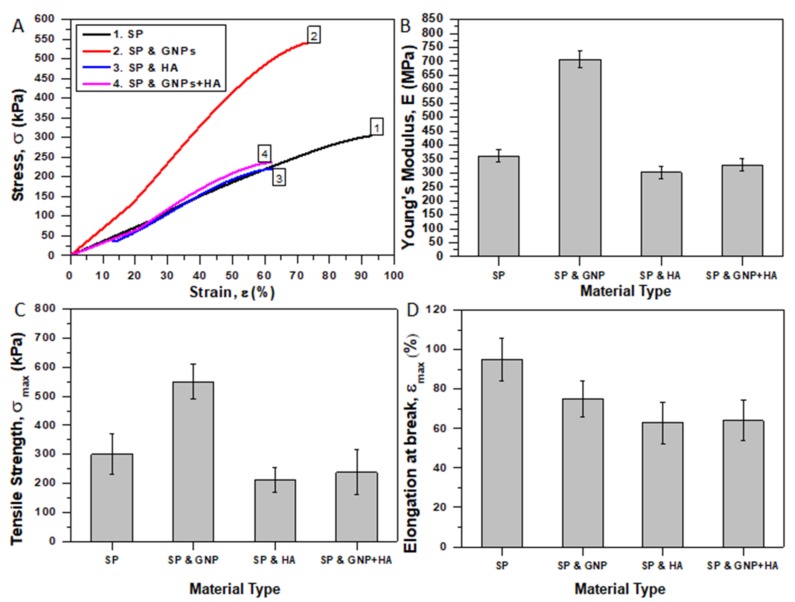
Results from tensile tests of pure and modified SP (with GNPs, HA, and mixture of them) electrospun scaffolds. (**A**) Representative stress (σ) vs. strain (ε) % curves, (**B**) Young’s modulus (E), (**C**) tensile strength (σ_max_), and (**D**) elongation at break (ε_max_) %. For all tensile properties standard deviations (SSD) are provided.

Figure 7A shows representative stress (σ) vs. strain (ε) % curves for the reference (pure SP) and modified scaffolds (with GNPs, HA, and mixture of them). For each specimen, the apparent E, σ_max_, and ε_max_ (%) values were calculated (Figure 7B–D). All scaffold types showed typical yield behavior. Yielding becomes more visible for scaffolds containing only HA while for scaffolds containing GNPs the yielding becomes hardly discernable (especially for modified ones with GNPs). An analogous behavior was also observed in Gkermpoura’s et al. work [26], in which Poly(methyl methacrylate) (PMMA) scaffolds were doped with GNPs. The bar chart of Figure 7B shows average values of E for all material sets. E values were calculated through linear regression analysis of the initial linear parts of stress-strain curves. The average values of E and σ_max_ for pure SP scaffolds were calculated to be 361.21 ± 21 MPa and 300.02 ± 70 kPa, respectively. Samples containing GNPs exhibited improved tensile properties by 95.6% (706.78 ± 29 MPa) and by 83.6% (550.97 ± 60 kPa) for the E and σ_max_ values, respectively. This behavior was also confirmed considering the experimental results of other investigations [26]. The high stiffness and high aspect ratio of the incorporated GNPs and their preferred orientation along the fiber axis seems to be beneficial for these scaffold types together with the reduction of the porogen fraction that was previously observed (see Figure 6B). On the other hand, samples containing HA exhibited slightly decreased tensile properties by 16.3% (302.31 ± 21 MPa) and by 29.3% (211.92 ± 43 kPa) for the E and σ_max_ values, respectively. Finally, samples containing the mixture of both filler types exhibited slightly reduced tensile properties if compared to the reference ones (reduced by 8.7% and 20.83% for the E and σ_max_ values, respectively), but were slightly enhanced if compared to the HA-modified ones (9% and 12% increase for the E and σ_max_ values, respectively) due to the GNP incorporation. For these samples, the E and σ_max_ values were calculated to be 329.60 ± 22 MPa and 237.52 ± 52 kPa, respectively. The reason why the mechanical properties were reduced is twofold: (a) due to the extremely average fiber diameter decrease (see Figure 2) and (b) due to the slight increase of final scaffolds’ porosity that was previously reported in paragraph 3.3. In similar study, HA was also responsible for the degradation of the mechanical properties [53]. It is also of note that the ε_max_ reduces by modification with samples containing HA to present the earliest failure. More precisely, the ε_max_ value was calculated to be 95, 75, 63, and 64% for pure SP and modified ones with HA, GNPs, and thee mixture of them, respectively. To summarize, the trend reveals a noteworthy increase of the apparent mechanical properties for scaffolds containing GNPs as they almost doubled, while by HA modification the properties of the final scaffold deteriorated.

## 4. Conclusions

A new matrix-type scaffold was successfully fabricated through electrospinning in order to support three-dimensional tissue formation for future biomedical applications. More precisely, a SP that is based on UPy hydrogen bonding units was utilized and appropriately modified with fillers (GNPs, HA, and mixture of them) for the needs of the current work. Pure and modified scaffolds were fabricated and were further utilized to investigate its structural, physical, and mechanical properties. According to SEM examinations, it was shown that by modification scaffolds with thinner average fiber diameters were obtained with HA-modified ones to exhibit the lower values. This behavior was found out to be attributed to the significant increase of the solutions’ electrical conductivities (especially for those containing HA) that led to enhanced bending instability during the electrospinning process. In addition, scaffolds’ doping made the surface to be more hydrophilic and to decrease the average pore area. The porogen fraction was slightly decreased for scaffolds containing GNPs, retained at the same levels for HA-modified ones, while slightly increased for GNP- and HA-modified scaffolds. Finally, SP scaffolds significantly enhanced the apparent tensile properties by GNP modification, while the presence of the HA led to their deterioration. In future work, the fabricated SP scaffolds will be further studied as potential candidate for tissue fabrication.

## Abbreviations

SP	Supramolecular polymer
UPy	Ureiodypyrimidone
GNPs	Graphene nanoplatelets
HA	Hydroxyapatite
ECM	Extracellular matrices
PLGA	Poly(lactic-co-glycolicacid)
T_g_	Glass transition temperature
FA	Formic acid
RT	Room temperature
SEM	Scanning electron microscopy
SE	Scanning electron
TEM	Transmission electron microscopy
A	Pore area
P	Porosity
M_1_	Mass of a fully compacted sample
M_2_	Mass of an electrospun sample (scaffold)
E	Young’s modulus
σ_max_	Ultimate tensile strength
ε_max_	Elongation at break
PMMA	Poly(methyl methacrylate)
